# Tumor Necrosis Factor (TNF) Is Required for Spatial Learning and Memory in Male Mice under Physiological, but Not Immune-Challenged Conditions

**DOI:** 10.3390/cells10030608

**Published:** 2021-03-09

**Authors:** Leda Mygind, Marianne Skov-Skov Bergh, Vivien Tejsi, Ramanan Vaitheeswaran, Kate L. Lambertsen, Bente Finsen, Athanasios Metaxas

**Affiliations:** 1 Institute of Molecular Medicine, Department of Neurobiology, University of Southern Denmark, J.B. Winsløws Vej 25, DK-5000 Odense C, Denmark; labbasowa@health.sdu.dk (L.M.); vivien.tejsi92@gmail.com (V.T.); ramanan1992@hotmail.com (R.V.); klambertsen@health.sdu.dk (K.L.L.); 2 BRIDGE—Brain Research Inter-Disciplinary Guided Excellence, Department of Clinical Research, University of Southern Denmark, J.B. Winsløws Vej 19, DK-5000 Odense C, Denmark; 3 Department of Forensic Sciences, Division of Laboratory Medicine, Oslo University Hospital, Loviseberggata, 60456 Oslo, Norway; rmmabe@ous-hf.no; 4 Department of Neurology, Odense University Hospital, J.B. Winsløws Vej 4, DK-5000 Odense C, Denmark; 5 School of Science, Department of Life Sciences, European University Cyprus, 6 Diogenis Str., Nicosia 1516, Cyprus

**Keywords:** TNF, LPS, inflammation, sickness behavior, aging, cognition, Barnes maze

## Abstract

Increasing evidence demonstrates that inflammatory cytokines—such as tumor necrosis factor (TNF)—are produced at low levels in the brain under physiological conditions and may be crucial for synaptic plasticity, neurogenesis, learning and memory. Here, we examined the effects of developmental TNF deletion on spatial learning and memory using 11–13-month-old TNF knockout (KO) and C57BL6/J wild-type (WT) mice. The animals were tested in the Barnes maze (BM) arena under baseline conditions and 48 h following an injection of the endotoxin lipopolysaccharide (LPS), which was administered at a dose of 0.5 mg/kg. Vehicle-treated KO mice were impaired compared to WT mice during the acquisition and memory-probing phases of the BM test. No behavioral differences were observed between WT and TNF-KO mice after LPS treatment. Moreover, there were no differences in the hippocampal content of glutamate and noradrenaline between groups. The effects of TNF deletion on spatial learning and memory were observed in male, but not female mice, which were not different compared to WT mice under baseline conditions. These results indicate that TNF is required for spatial learning and memory in male mice under physiological, non-inflammatory conditions, however not following the administration of LPS. Inflammatory signalling can thereby modulate spatial cognition in male subjects, highlighting the importance of sex- and probably age-stratified analysis when examining the role of TNF in the brain.

## 1. Introduction

Cytokines are a class of immunity-associated molecules that have profound effects on the brain’s response to peripheral inflammation [1]. In addition, inflammatory cytokines in the central nervous system (CNS) have been recognized as an integral part of neurologic and psychiatric disorders, including schizophrenia, affective disorders, stroke, and chronic neurodegenerative and neuroinflammatory diseases [2,3,4,5,6]. While the detrimental role of inflammation in the CNS is being increasingly scrutinized, research on the involvement of pro-inflammatory cytokines in physiological processes, such as learning and memory, is rather scarce. Increasing evidence suggests that cytokines that were once considered purely inflammatory, such as tumor necrosis factor (TNF), are produced in the brain under physiological conditions, and may be crucial for synaptic development and regulation [7,8,9,10,11,12]. 

Within the CNS, TNF is primarily produced by the myeloid-derived microglia, and in the case of neuroinflammation, also by infiltrating blood-borne immune cells [9,13,14,15,16]. Studies examining the role of TNF in hippocampal-dependent learning and memory under physiological, non-inflammatory conditions have produced conflicting results. In the Morris water maze test (MWMT) and the Barnes maze test (BMT), mice with genetic ablation of the TNF protein (TNF knockout, TNF-KO) have been shown to be both compromised [2,7] and cognitively normal [17,18]. In addition, the effects of TNF on cognitive behavior might be age-specific, as TNF-KO mice are impaired in the BM compared to wild-type (WT) animals at 3, but not 6 or 12 months of age [19]. Unlike the disparities observed in studies with TNF-KO mice, TNF-overexpressing mice, with (Tg6074) or without (TgK3) neurological alterations, are impaired in the MWMT, implying that TNF-driven neuroinflammation might be more consistently associated with cognitive impairment [20]. These observations, however, may be confounded by the decreased motor activity of TNF-overexpressing mice compared to WT animals. 

Lipopolysaccharide (LPS) is an immunostimulatory glycolipid located on the outer membrane of all Gram-negative bacteria. It binds to Toll-like receptor 4 (TLR4) in conjunction with CD14, MD-2 and LPS-binding protein, which are all expressed by the microglia [21,22]. Several studies have shown that peripheral administration of LPS impairs spatial memory in rodents [23,24,25,26,27]. However, the effects of LPS are strongly sex- and dosage-dependent, and it has been suggested that LPS-induced cognitive impairment can be alternatively explained by changes in the general state of the animals’ well-being [28]. Indeed, LPS administration is known to produce sickness behavior in a time- and dose-dependent manner, especially in male mice [29,30], which can interfere with measurements of cognition in rodents.

The aim of the present study was to examine the role of TNF in spatial cognition under physiological conditions and following LPS-induced inflammation. Based on accumulating data on the homeostatic functions of TNF, we reasoned that TNF-KO mice would be impaired in the BMT compared to WT animals. To examine whether TNF impairs cognition after peripheral immune activation, we first characterized the effects of 0.5 mg/kg LPS on sickness behavior and locomotor activity using both male and female mice. TNF-KO and WT mice were then studied in the BM at a time-point when LPS-induced sickness behavior had dissipated, to avoid potential confounding.

## 2. Materials and Methods

### 2.1. Animals and Treatment

All procedures complied with Danish law (Bekendtgørelse af lov om dyreforsøg, LBK nr 253 af 08/03/2013) and European Union directive 2010/63/EU, regulating animal research. Ethical permission was granted by the Animal Experimental Council (nr 2016/15-0201-00952).

TNF-KO mice were originally purchased from the Jackson laboratories [31]. The animals were bred and maintained on a C57BL/6J background in the Biomedical Laboratory of the University of Southern Denmark, under controlled temperature, humidity and light conditions (lights on: 7 am; 12:12 h light-dark cycle). WT littermate and non-littermate C57BL6/J mice were used as controls. Food and water were available ad libitum. A total of 73, group-housed (*n* = 4–8/cage), 11–13-month-old, male and female mice were employed in the studies.

At least 1 week prior to experimentation, animals were transported and housed in a dedicated behavioral testing facility. Experiments were then conducted during the light phase of the light/dark cycle, between 08:00 h and 15:00 h. LPS (*E. coli* serotype 0111:B4, Sigma-Aldrich, Søborg, Denmark) was dissolved in sterile, phosphate-buffered saline (PBS), and administered once intraperitoneally (IP) to TNF-KO and WT mice at the dose of 0.5 mg/kg. Vehicle mice received PBS in a final injection volume of 150 µL. 

### 2.2. Assessment of Non-Cognitive Effects

The effects of 0.5 mg/kg LPS on sickness behavior and locomotor function in the open field test were examined using both male and female mice (*n* = 4/group).

#### 2.2.1. Sickness Behavior 

Immediately after the IP injection of LPS and PBS, mice were placed into individual observation chambers and monitored for sickness behavior by two trained observers, blinded to the animals’ genotype and treatment group. Sickness behavior was scored on a three-point scale (1–3) with symptoms of ptosis (drooping eyelids), lethargy (curled body posture and diminished locomotion after single prompting), and piloerection (ruffled fur, piloerection at the nape of the neck) included in the score [32]. Each symptom present received 1 point, resulting in a highest possible score of 3. Sickness behavior was scored for 6 h, at 30 min intervals for the initial 4 h and at 1 h intervals subsequently. Thereafter, mice were returned to their home cages and re-evaluated for sickness behavior 24 h after the injections. Animal weights were registered before and 24 h after the injections. 

#### 2.2.2. Open Field

The open field test was performed 24 h after injections, immediately after the final evaluation of sickness behavior. Animals were placed in the center of a non-transparent, square polypropylene arena (W 45 cm × D 45 cm × H 40 cm), which was illuminated by four 120 W halogen spotlights. Spontaneous locomotor activity was tracked for 10 min, by using an overhead video camera (SSC-DC3578P, Biosite, Stockholm, Sweden) connected to SMART video-tracking software (Panlab, Barcelona, Spain; [33,34]). For analysis purposes, the arena was digitally divided into 2 zones: center (W 20 cm × D 20 cm) and periphery. Total distance travelled and the time-spent (s) in the center and periphery of the arena were recorded for each mouse. After each test, the arena was cleaned with 70% ethanol, followed by cleaning with a damp cloth, to remove odors. 

### 2.3. Determination of TNF mRNA

Animals were euthanized by cervical dislocation immediately following the open-field tests. The left hippocampi were isolated and frozen in dry ice for measurement of TNF mRNA by reverse transcriptase polymerase chain reaction (RT PCR), as described previously [35]. Briefly, Trizol^®^-isolated RNA (2 μg) from the hippocampus of WT, PBS and LPS-treated male and female mice was reverse-transcribed to cDNA, by using the Applied Biosystems™ high-capacity cDNA transcription kit (ThermoFisher Scientific Inc., Roskilde, Denmark). Samples were analyzed in triplicates on a StepOnePlus™ Real-Time PCR system (Applied BiosystemsTM, ThermoFisher Scientific Inc., Roskilde, Denmark). Each 20 μL sample contained nuclease-free H_2_O (ThermoFisher Scientific Inc., Roskilde, Denmark), 1× Maxima SYBR Green dye for TNF or 1× TaqMan Probe master mix for hypoxanthine phosphoribosyl transferase (HPRT1; ThermoFisher Scientific Inc., Roskilde, Denmark), 500 nM forward and reverse primers (TAG Copenhagen A/S, Copenhagen, Denmark), undiluted cDNA for TNF or 10× diluted cDNA for HRPT1 (reference gene). Primer sequences for HPRT1 were: forward, 5΄-GTT AAG CAG TAC AGC CCC AAA ATG-3΄; reverse, 5΄-AAA TCC AAC AAA GTC TGG CCT GTA-3΄ and for TNF: forward, 5΄-TGG CCT CCC TCT CAT CAG TTC-3΄; reverse, 5΄-ACT TGG TGG TTT GCT ACG ACG-3΄. For each sample, relative fold differences from the mean TNF levels of unmanipulated control samples were calculated after normalisation of TNF against HPRT1 mRNA, by using the ΔΔCt method. Nuclease-free H_2_O and genomic DNA instead of cDNA were used for control purposes.

### 2.4. Assessment of Cognitive Effects

The Barnes maze test (BMT) is a dry-land alternative to the MWMT and uses bright light instead of water for motivating behavior. In this study, the BM arena was an open, circular platform (92 cm), possessing 20 holes that were evenly distributed around its perimeter (Panlab, Barcelona, Spain). The arena was situated 1.5 m above the ground and illuminated by a set of four bright lights (4 × 120 W). A recessed dark box was placed under one of the twenty holes, providing animals with a route to escape from the brightly lit arena.

Male, age-matched, WT and TNF-KO mice were tested in the BM in a paired manner. The animals were injected once with PBS or 0.5 mg/kg LPS, 48 h before the spatial acquisition (learning) phase of the BMT (*n* = 7–8/group). The 48 h time-point, as well as the gender of the animals used for LPS injections, were chosen based on the dose-assessment studies, characterizing the effects of 0.5 mg/kg LPS on sickness behavior and locomotor activity in the open field. Data were generated after four independent BM experiments, which were performed and analyzed in a blinded manner. The location of the escape box was kept constant during a given experiment but was changed to different positions between experiments. In addition to male mice, a set of female WT and TNF-KO mice was also tested in the BM under identical conditions, 48 h following an injection of PBS only (*n* = 4–7/group).

Mice were habituated to handling and novelty for a period of five days prior to the BMT. The BMT was then performed according to Sunyer et al. 2007 [36]. On DAY 0, animals were adapted to the BM arena. During this single session, mice were placed in a transparent tube in the middle of the maze. Following 30 s, the animals were guided towards the escape hole on a time frame between 10–15 s and allowed 2 min to climb down and enter the escape box. When mice did not enter the escape box after 2 min, they were gently pushed inside, by using the transparent tube. After entering the dark escape box, mice were allowed inside for a period of 2 min. Following this adaptation session, animals were returned to their home cage, and the BM arena cleaned as described for the open field test. Spatial learning took place on DAYS 1, 2 and 3. During the acquisition phase, each mouse was placed within a non-transparent starting cylinder in the center of the arena for 15 s (starting point randomization). The lights were then turned on, the tube lifted, and the animal was allowed 3 min to locate the dark escape box. If the mouse located and climbed inside the box, it was kept inside for 1 min. If the mouse did not find the box within 3 min, it was gently guided toward the box and placed inside using the transparent tube. The animals received 4 training trials, each lasting 180 s, on 3 consecutive days, all sessions recorded by camera. After each trial, the maze and escape box were cleaned thoroughly. The memory-testing, probe trials were conducted 24 h and 7 days after the end of the acquisition phase. The escape box was removed for these sessions. Mice were placed in the non-transparent tube for 15 s, the lights turned on, and the animals allowed 90 s to explore the maze. 

Measurements during the BM experiments included total latency, which was the time required for an animal to enter the escape box (all 4 paws inside the box), primary latency, which was the time required for an animal to reach the escape hole for the first time (first correct nose-poke), target block time, which was measured on DAYS 4 & 11 and was defined as the time each mouse spent in the quarter of the arena where the escape box used to be situated, and nose-poke distribution, which was the number of nose-pokes conducted by each animal in each of the 20 holes on DAYS 4 and 11. Immediately after the BMT, mice were euthanized by cervical dislocation and the left hippocampi isolated and frozen in dry ice. Samples from male mice were subsequently sent to Oslo University Hospital, Norway, where they were assayed for total glutamate and noradrenaline content. 

### 2.5. Determination of Neurotransmitters 

Determination of neurotransmitters was performed using a previously described ultra-high-performance liquid chromatography tandem mass spectrometry (UHPLC-MS/MS) method by Bergh et al. [37] with minor modifications. Stock solutions of L-noradrenaline hydrochloride (Merck KGaA, Darmstadt, Germany) and L-glutamic acid (Merck), and the internal standards noradrenaline-d_6_ hydrochloride (C/D/N Isotopes Inc., Quebec, PQ, Canada) and DL-glutamic acid-d_3_ (C/D/N Isotopes Inc.), were prepared in 25 mM formic acid and stored at 4 °C. Working solutions for calibrators and quality control samples were prepared independently by dilution of stock solutions in 25 mM formic acid. Calibrators (10–4000 nm) and quality control samples (30–3200 nm) were prepared by appropriate dilution of the working solutions in 25 mM formic acid.

Hippocampal samples were homogenized in type 1 water (50 mg/mL) using a VirSonic 300 ultra sound sonicator (VirTis, New York, NY, USA). Aliquots were collected for triplicate determinations of total protein content [38], prior to sample preparation for UHPLC-MS/MS analysis. Absorbance was measured (750 nm) using a Hidex Sense Microplate Reader (Hidex, Turku, Finland). 

For determination of neurotransmitters, brain homogenate (50 μL) and internal standard (25 µL) were added to brown eppendorf tubes. Ice-cold 250 mM formic acid (50 µL) was added previous to vortexing. The samples were centrifuged before the supernatant was transferred to autosampler vials. For calibrators and quality control samples, solution (50 μL) was added to internal standard (25 µL) and 225 mM formic acid (50 μL).

Analysis of mouse hippocampus samples was performed using an Acquity UHPLC system (Waters, Milford, MA, USA) coupled to a Xevo-TQ triple quadrupole mass spectrometer with an electrospray ionization interface (Waters). Chromatographic separation of the analytes was performed using an Acquity HSS T3 column (2.1 × 100 mm, 1.8 µm particles; Waters, Wexford, Ireland) at 65 °C with a mobile phase consisting of 25 mM formic acid (solvent A) and methanol (solvent B) at a flow rate of 0.5 mL/min. The separation was carried out using a 5.3 min gradient profile. Mass spectrometric analyses were carried out using positive ionization with the quadrupoles operating in the multiple reaction monitoring mode, with 2 transitions for noradrenaline (170.2 > 152.1 and 170.2 > 107.1) and glutamic acid (148.0 > 101.9 and 148.0 > 84.0), and one transition for the internal standards noradrenaline-d_6_ (176.2 > 158.1) and DL-glutamic acid-d_3_ (151.0 > 87.0). The limit of quantification for the analytes was between 100 and 200 nm. Data was acquired and processed using Masslynx™ 4.1 software (Waters, Milford, MA, USA). For more detailed information about the UHPLC-MS/MS analysis, see Bergh et al. [37]. 

### 2.6. Statistical Analysis

Sickness behavior was compared between groups using non-parametric statistics (Mann–Whitney *U* tests). Results are presented as the median ± IQR (Q3-Q1) of *n* = 4 animals per group. Total distance travelled in the open field and weight loss were analyzed by three-way analysis of variance (ANOVA), for the independent factor treatment (LPS vs. PBS), gender (male vs. female) and genotype (WT vs. TNF-KO). TNF mRNA levels were analyzed in the hippocampus of PBS- and LPS-treated, male and female mice using two-way ANOVA. Behavior in the BMT was analyzed by mixed model ANOVA, with time (DAY) as the within-subject, repeated variable, and genotype and treatment as the between-subject, independent variables. Data are presented as mean ± standard error of the mean (SEM) of *n* = 7–8 animals per group in the case of male mice and *n* = 4–7 in the case of female mice. Neurotransmitter levels in the hippocampus of male mice were analyzed using two-way ANOVA, for the factors genotype and treatment. In all cases, significant main and interaction effects (*p* < 0.05) were further analyzed by Bonferroni post hoc tests. The analysis was performed with STATISTICA (v10; StatSoft Inc., Tulsa, OK, USA) and GraphPad Prism software (v8.4.3; GraphPad Holdings, San Diego, CA, USA).

## 3. Results

### 3.1. Assessment of Non-Cognitive Effects of LPS Administration

**Figure 1:**
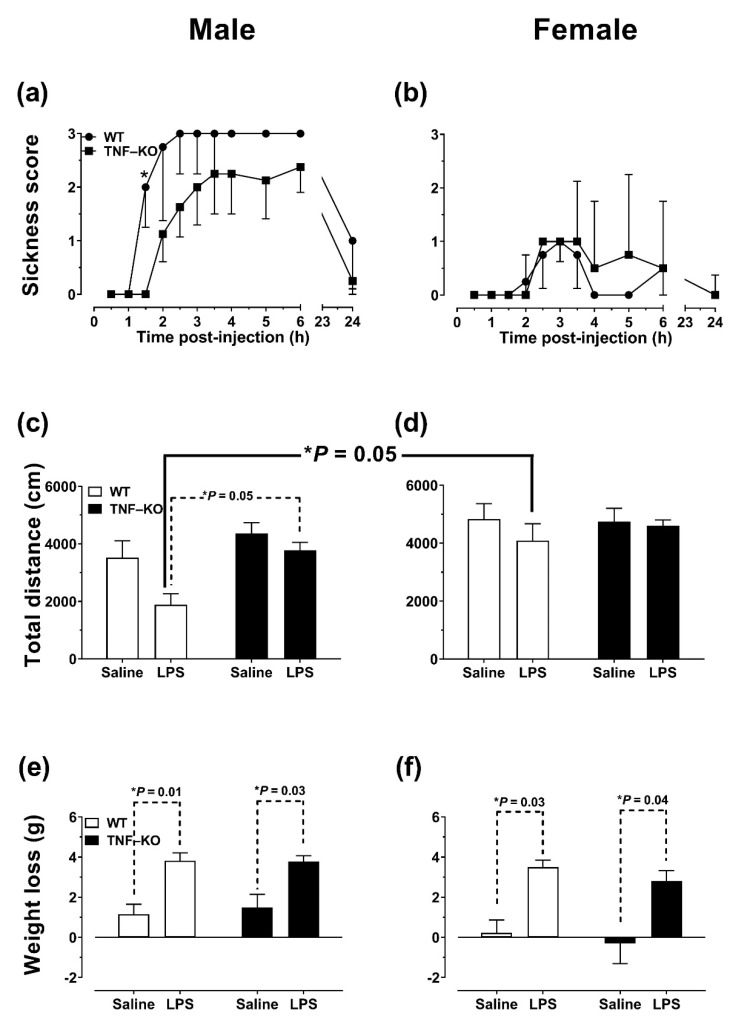
Non-cognitive effects of systemic LPS administration. The effects of 0.5 mg/kg LPS on sickness behavior (**a**,**b**), total distance travelled (**c**,**d**) and weight loss (**e**,**f**) were evaluated 24 h post-injection in male and female WT and TNF-KO mice. (**a**,**b**) Male mice were more susceptible to LPS-induced sickness behavior compared to females. TNF deletion reduced symptom score in male mice (**a**), an effect that was not observed in females (**b**). Results are presented as the median score ± IQR of *n* = 4 mice per group, and were analyzed using non-parametric, Mann–Whitney *U* tests. In (**a**), * *p* < 0.05 vs. TNF-KO at 1.5 h. (**c**,**d**) In male mice (**c**), LPS treatment reduced total distance travelled in WT, but not TNF-KO mice, an effect that was not observed in female mice (**d**). Moreover, male, LPS-treated WT mice travelled less than their female counterparts. (**e**,**f**) Weight was reduced in both male (**e**) and female mice (**f**), irrespective of genotype. Results in **c**–**f** are presented as the mean ± SEM of *n* = 4 mice per group and were analyzed using three-way ANOVA and Bonferroni post hoc tests.

To control for potential confounders of cognitive performance after systemic immune activation, we evaluated the effects of 0.5 mg/kg LPS on sickness behavior and locomotion using both male and female mice (Figure 1). In male mice (Figure 1a), the deletion of TNF decreased sickness score compared to WT animals (*U* = 647.0, *p* < 0.01), an effect not observed in female mice (Figure 1b; *U* = 946.5, *p* > 0.05) (Mann–Whitney *U* tests). Thus, the median sickness score for the 24 h observation period was 3 for male WT mice (IQR = 1.3), 2.25 for male TNF-KO mice (IQR = 2.1), 0 for female WT (IQR = 0.5), and 0.25 for female TNF-KO mice (IQR = 1.0). In addition to presenting with fewer symptoms, male TNF-KO mice showed a delayed reaction to LPS-induced sickness behavior compared to WT animals. LPS-induced symptoms peaked at 2.5 h and 3.5 h post-injection in male WT and TNF-KO animals, respectively. Symptoms of sickness behavior were evident in 50% of male WT mice 24 h after the administration of 0.5 mg/kg LPS. Mann–Whitney *U* tests, conducted for each time-point individually, revealed significantly increased sickness score in male WT vs. TNF-KO mice at 1.5 h post-injection (*U* = 0.0, *p* < 0.05). Moreover, males displayed more sickness behavior compared to females, regardless of genotype (WT male vs. female: *U* = 396.5, *p* < 0.001; TNF-KO male vs. female: *U* = 709.5, *p* < 0.05; Figure 1a,b).

Mice were tested in the open field immediately following the evaluation of sickness behavior (Figure 1c,d). The genetic deletion of TNF did not alter spontaneous locomotor activity in PBS-treated male (Figure 1c) and female mice (Figure 1d). In males, LPS treatment reduced total distance travelled in WT mice by >50% compared to PBS control (*p* = 0.10) and LPS-treated TNF-KO mice (*p* < 0.05; Bonferroni post hoc tests) (Figure 1c). In females, no between-group differences were observed (*p* > 0.05) (Figure 1d). In addition, Bonferroni post hoc tests showed that LPS-treated WT males had lower locomotor activity compared to their female counterparts (Figure 1c,d; solid line). In all groups, time-spent in the periphery of the open field exceeded 90% of total test time. Three-way ANOVA confirmed that total distance travelled was lower in male vs. female (F_(1,24)_ = 14.0, *p* < 0.001), WT compared to TNF-KO (F_(1,24)_ = 6.3, *p* = 0.02), and LPS- compared to PBS-treated mice (F_(1,24)_ = 6.1, *p* = 0.02).

Mice were weighed right before and 24 h after the IP administration of PBS or LPS. As illustrated in Figure 1e,f, LPS-injected animals lost significantly more weight compared to control mice (F_(1,24)_ = 46.9; *p* < 0.001), a reduction that was observed in both WT and TNF-KO animals, irrespective of genotype (F_(1,24)_ = 0.3; *p* > 0.05). There was an overall effect of gender on weight loss (F_(1,24)_ = 5.9, *p* = 0.02), which did not reach statistical significance at post hoc testing.

### 3.2. Hippocampal Levels of TNF mRNA 

**Figure 2:**
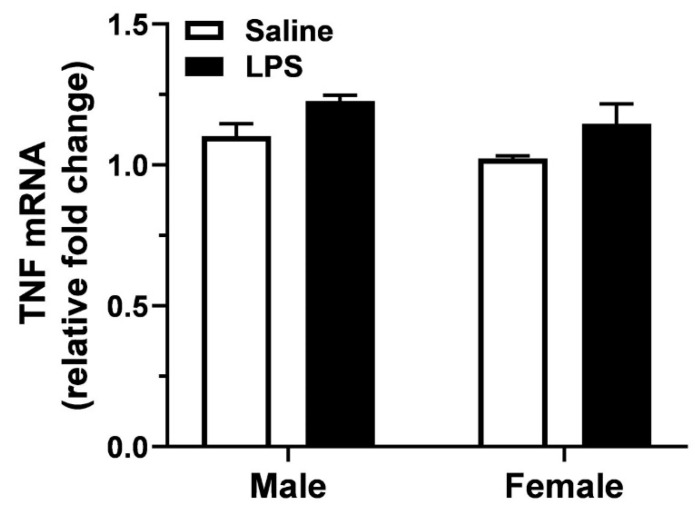
Hippocampal TNF mRNA levels, 24 h following the administration of LPS. There was an overall effect of treatment on the levels of TNF mRNA expression by two-way ANOVA, which did not reach significance in male and female mice (*p* > 0.05, Bonferroni post hoc tests). Results are the mean ± SEM of *n* = 3–4 animals per group. Mice survived for 24 h after the injection of 0.5 mg/kg LPS.

Mice were killed by cervical dislocation immediately after the open-field tests, 24 h following the injection of 0.5 mg/kg LPS. TNF mRNA levels in the hippocampus were overall higher in LPS-treated mice compared to vehicle (Figure 2, treatment effect: F_(1,10)_ = 9.5, *p* = 0.01; two-way ANOVA). However, the increase was small, and individual post hoc comparisons showed no differences in TNF mRNA between PBS- and LPS-treated, male (*p* > 0.05) and female mice (*p* > 0.05). In addition, there was a tendency of increased TNF mRNA in male vs. female subjects, which did not reach statistical significance at the 24 h time-point (sex effect: F_(1,10)_ = 3.9, *p* = 0.07). Two-way ANOVA showed no significant sex × treatment interaction effects on hippocampal TNF mRNA (F_(1,10)_ = 0.0, *p* > 0.05). 

### 3.3. Impaired Learning in PBS-Treated Male TNF-KO Mice during the Acquisition Phase of the BMT

To maximize the probability of detecting cognitive effects of developmental TNF deletion, the BMT was conducted using male mice, which were more susceptible to LPS than females in the sickness-assessment experiments. The acquisition phase of the BMT was initiated 48 h after the injection of PBS and LPS to WT and TNF-KO mice. At this time-point, LPS-treated animals showed no symptoms of sickness behavior (score = 0).

**Figure 3:**
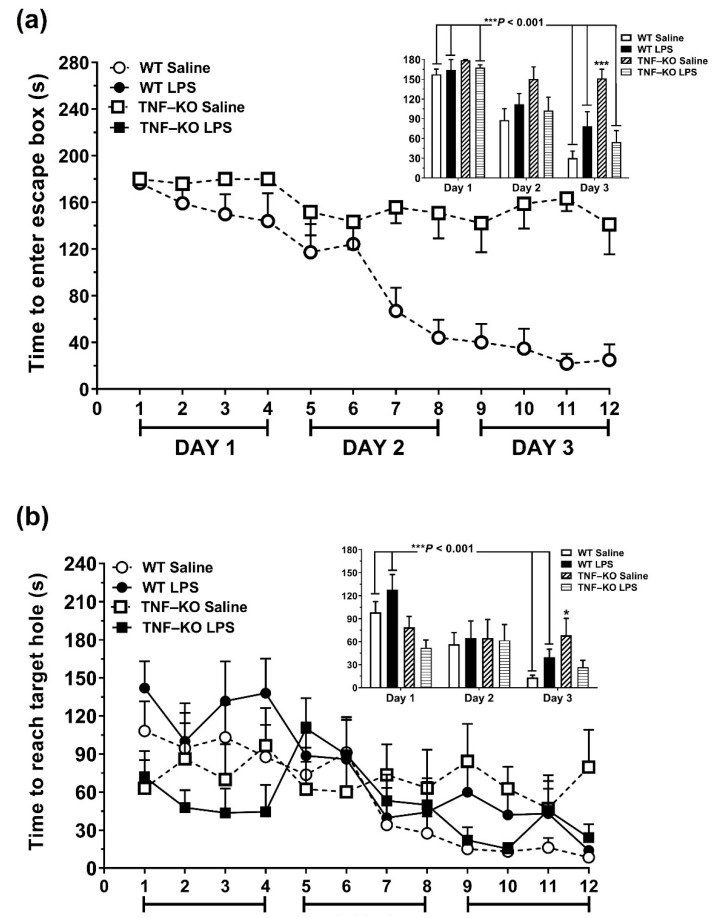
Acquisition phase of the BMT in LPS- and PBS-treated WT and TNF-KO male mice. The acquisition phase consisted of 4 training sessions daily and was conducted over 3 consecutive days. (**a**) Progressive reductions in the time required to enter the escape box (total latency) were observed in all groups, except for PBS-treated TNF-KO mice. The insert shows that PBS-treated KO mice had the longest total latency times vs. all other groups on DAY 3 (*** *p* < 0.001, Bonferroni post hoc test). (**b**) The time required to reach the escape box for the first time (primary latency) was reduced in a time-dependent manner primarily in WT, rather than TNF-KO mice. On DAY 3, primary latency was longer in PBS-treated TNF-KO vs. PBS-treated WT mice (* *p* < 0.05, Bonferroni post hoc tests). Results are the mean ± SEM of *n* = 7–8 animals per group and were analyzed using a mixed-model ANOVA and Bonferroni post hoc *tests*.

For total latency (Figure 3a), the time required to enter the escape box was shorter on DAY 3 vs. DAY 1 in both PBS- and LPS-treated WT mice (*p* < 0.001, Bonferroni post hoc tests; insert Figure 3a). This time-dependent reduction was not observed in PBS-treated TNF-KO mice (DAY 3 vs. DAY 1, *p* > 0.05). In LPS-injected TNF-KO animals, total latency was reduced on DAY 3 vs. DAY 1 (*p* < 0.001), similar to what was observed for WT mice. 

On DAY 3, time to enter the escape box was significantly longer in PBS-treated TNF-KO mice compared to all other groups (*p* < 0.001, Bonferroni post hoc tests). A mixed model ANOVA confirmed significant main effects of time (F_(2,52)_ = 57.3, *p* < 0.001) and genotype (F_(2,52)_ = 6.3, *p* < 0.02), as well as significant treatment × genotype (F_(2,52)_ = 11.4, *p* < 0.01) and time × treatment × genotype (F_(2,52)_ = 7.4, *p* = 0.001) interaction effects on the total latency of WT and TNF-KO mice. 

For primary latency (Figure 3b), time to reach the escape hole for the first time was reduced in a time-dependent manner primarily in WT, rather than TNF-KO mice (genotype x time interaction effect: F_(2,52)_ = 8.6, *p* < 0.001; insert Figure 3b). Two-way ANOVA, conducted for each day individually, showed that TNF-KO mice had shorter primary latencies compared to WT animals on learning DAY 1 (genotype effect: F_(1,26)_ = 10.7, *p* < 0.01). By DAY 3, however, this trend had almost reversed (genotype effect: F_(1,26)_ = 2.9, *p* = 0.10), and PBS-injected KO mice now took longer to reach the escape hole compared to their WT counterparts (genotype × treatment interaction: F_(1,26)_ = 7.5, *p* = 0.01; *p* < 0.05, Bonferroni post hoc tests) (Figure 3b). 

### 3.4. Memory Deficits in PBS-Treated Male TNF-KO Mice during the Probe Phase of the BMT 

The escape box was removed for the memory-probing phase of the BMT. The time-spent in the quarter of the arena where the escape box used to be situated (target block time) was measured as an index of spatial memory, 24 h (DAY 4) and 7 days (DAY 11) following the end of the acquisition phase. Primary latency and the distribution of nose-pokes throughout the arena were also measured. 

**Figure 4:**
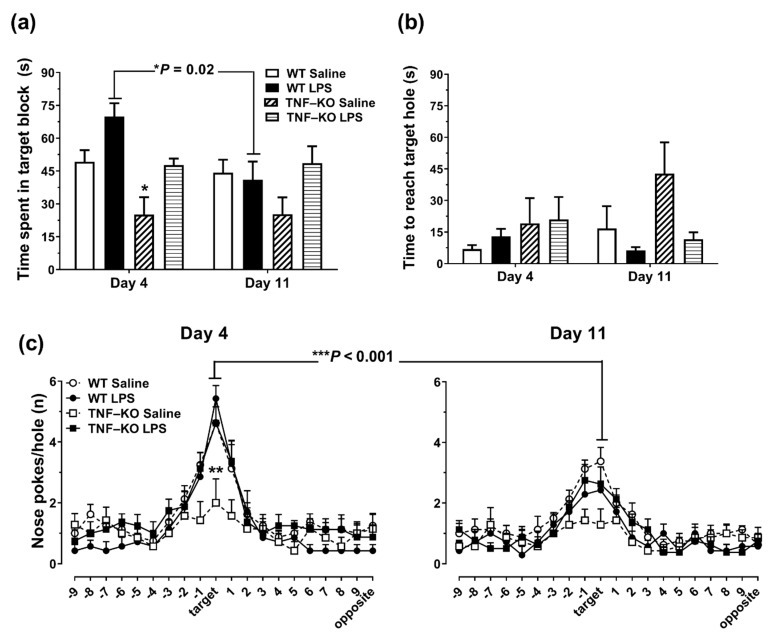
Memory-probing phase of the BMT in LPS- and PBS-treated WT and TNF-KO male mice. The escape box was removed for the probe sessions, which were conducted 24 h (DAY 4) and 7 days (DAY 11) following the acquisition phase. (**a**) On day 4, PBS-treated TNF-KO mice spent less time in the quarter of the arena where the escape box used to be situated (target block) compared to PBS-treated WT (* *p* < 0.05) and LPS-treated TNF-KO animals (* *p* < 0.05). Target block time was reduced in LPS-treated WT mice on DAY 11 vs. DAY 4 whereas PBS-treated WT mice had similar target block times on DAY 4 and DAY 11. (**b**) For primary latency, there were no between-group differences during the probing phase of the BMT. (**c**) The distribution of nose pokes across the BMT arena was practically random in PBS-treated TNF-KO mice. On DAY 4, the number of nose-pokes at the escape hole was lower in PBS-treated TNF-KO mice compared to all other groups examined (** *p* < 0.01). On DAY 11 vs. DAY 4, there was a time-dependent reduction in the number of target hole nose-pokes in all groups, except for PBS-treated TNF-KO mice. Results are the mean ± SEM of *n* = 7–8 mice per group and were analyzed using repeated measures ANOVA and Bonferroni post hoc tests.

On DAY 4, PBS-treated TNF-KO mice spent less time in the target block compared to PBS-treated WT and LPS-treated TNF-KO mice (*p* ≤ 0.05, Bonferroni post hoc tests; Figure 4a). On DAY 11, there were no differences in target block time between groups. LPS-treated WT mice spent less time in the target block on DAY 11 vs. DAY 4 (*p* = 0.02, Bonferroni post hoc tests), an effect that was not observed in LPS-treated TNF-KO mice. Repeated measures ANOVA showed significant main effects of time (F_(1,26)_ = 5.0, *p* < 0.05), genotype (F_(1,26)_ = 6.9, *p* = 0.01) and treatment (F_(1,26)_ = 8.3, *p* < 0.01) on target block time, along with significant time x genotype interaction effects (F_(1,26)_ = 5.6, *p* < 0.05). For primary latency (Figure 4b), time to reach the escape hole was longer on DAY 11 vs. DAY 4 in PBS vs. LPS-treated animals (time x treatment interaction effect: F_(1,26)_ = 5.0, *p* < 0.01), irrespective of genotype (F_(1,26)_ = 3.5, *p* = 0.07; repeated measures ANOVA). However, the effects were small, and individual post hoc comparisons showed no between-group differences in primary latency during the probing phase of the BMT (Figure 4b). 

The distribution of nose-pokes across the BMT arena on DAYS 4 and 11 is shown in Figure 4c. On DAY 4, PBS-injected TNF-KO mice performed significantly less nose-pokes at the escape hole compared to all other groups of mice (*p* < 0.01; Bonferroni post hoc tests). In addition, the number of target-hole nose-pokes was reduced on DAY 11 vs. DAY 4 in all groups of mice (*p* < 0.001), except for PBS-treated TNF-KO mice (*p* > 0.05; Bonferroni post hoc tests). A mixed model ANOVA confirmed significant main effects of hole position (F_(19,988)_ = 41.3, *p* < 0.001) and time (F_(1,52)_ = 4.6, *p* < 0.05) on the distribution of nose-pokes, along with significant genotype × treatment (F_(1,52)_ = 5.9, *p* < 0.05), hole × treatment (F_(19,988)_ = 2.6, *p* < 0.001), hole × genotype (F_(19,988)_ = 2.7, *p* < 0.001) and hole × time interaction effects (F_(19,988)_ = 3.2, *p* < 0.001).

### 3.5. Female TNF-KO and WT Mice Are Not Different in the BMT 

**Figure 5:**
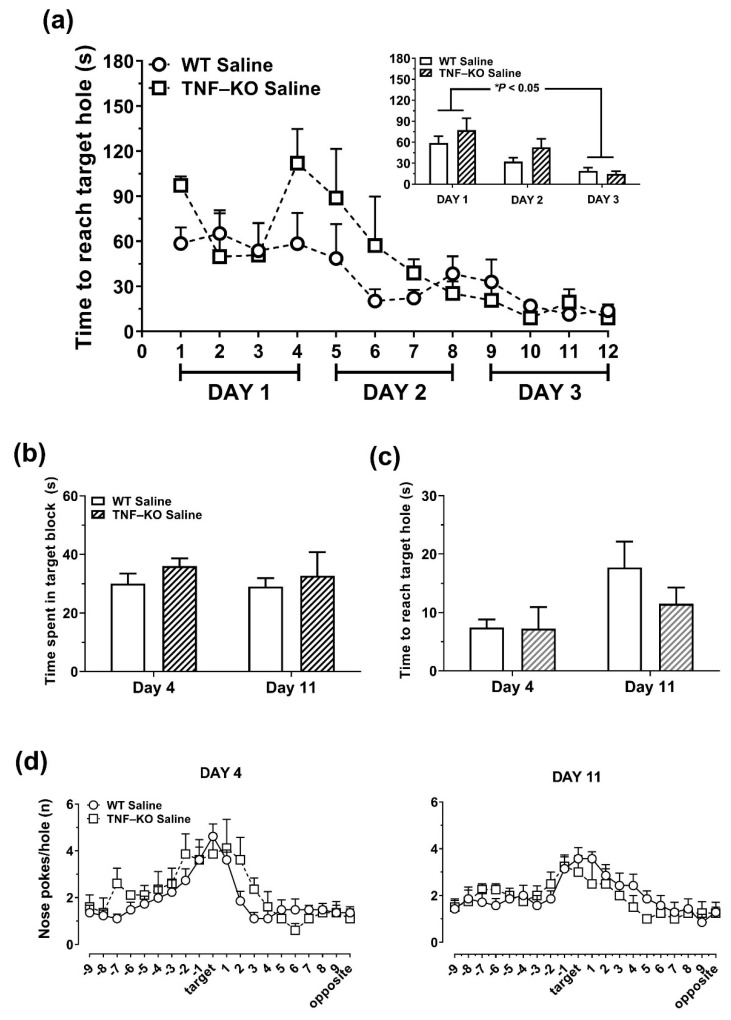
PBS-treated female TNF-KO mice are not impaired in the BMT. We examined whether the genetic deletion of TNF impairs BMT performance in female, PBS-treated, WT (*n* = 7) and KO mice (*n* = 4). (**a**) Primary latency was reduced on DAY 3 vs. DAY 1, in both WT and TNF-KO mice. (**b**,**c**) Time-spent in target block (**b**) and primary latency (**c**) were not different between groups during the memory probing sessions on DAYS 4 and 11. (**d**) There was no effect of genotype and time on the distribution of nose-pokes on DAYS 4 and 11.

Unlike male mice, PBS-treated female TNF-KO and WT animals were not different during the learning and memory-probing phases of the BMT (Figure 5). On learning DAYS 1-3 (Figure 5a), ANOVA showed significant main effects of time on primary latency (F_(2,18)_ = 14.1, *p* = 0.001), with no significant genotype (F_(1,9)_ = 3.2, *p* = 0.11) and genotype × time interaction effects (F_(2,18)_ = 1.0, *p* > 0.05). Moreover, there was no effect of genotype on target block time (Figure 5b; F_(1,9)_ = 1.6, *p* > 0.05), primary latency (Figure 5c; F_(1,9)_ = 1.3, *p* > 0.05), and nose-poke distribution on testing DAYS 4 and 11 in female animals (Figure 5d; F_(1,19)_ = 0.15, *p* > 0.05).

### 3.6. Neurotransmitter Levels in the Hippocampus of Male, PBS- and LPS-Treated Mice

**Figure 6:**
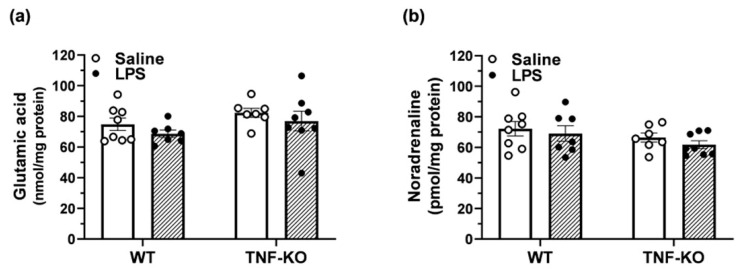
No change in neurotransmitter content in the hippocampus of male mice. The hippocampi of male WT and TNF-KO mice were isolated immediately after the end of the BMT. The levels of glutamate (**a**) and noradrenaline (**b**) were quantified by UHPLC-MS/MS. There were no differences in the level of neurotransmitters between groups. Results are the mean ± SEM of *n* = 7–8 animals per group.

To address how TNF deletion may impair the performance of male mice in the BMT, levels of neurotransmitters related to cognition, including glutamate and noradrenaline, were quantified in the hippocampus of WT and TNF-KO animals (Figure 6). Two-way ANOVA showed no effects of treatment (F_(1,26)_ = 1.7, *p* > 0.05) and genotype (F_(1,26)_ = 3.2, *p* = 0.09) on the levels of glutamate in the hippocampus (Figure 6a). Moreover, treatment (F_(1,26)_ = 1.0, *p* > 0.05) and genotype (F_(1,26)_ = 2.7, *p* > 0.05) had no effect on the hippocampal levels of noradrenaline in male mice (Figure 6b).

## 4. Discussion

This study was performed to examine the role of TNF in spatial learning and memory under physiological and immune-challenged conditions, by using the BMT and LPS administration for the induction of systemic inflammation. Our main finding is that TNF is required for spatial learning and memory in male mice under baseline conditions, but not following the administration of LPS. Moreover, the results indicate that the role of TNF in spatial cognition depends on sex, since female, PBS-treated WT and TNF-KO mice were not different in the BMT.

In our initial study, examining the non-cognitive responses to LPS at 0.5 mg/kg, sickness behavior was more pronounced in male, rather than female mice. This observation corroborates a large body of preclinical and clinical literature, showing differences in the susceptibility to endotoxemia between sexes [29,39,40]. In humans, peripheral blood mononuclear cells (PBMC) produce more TNF and IL-1β following incubation with LPS in males vs. females [41,42,43]. Additionally, neutrophils from human males produce higher levels of TNF compared with females when stimulated with LPS and express more TLR4 following stimulation with IFN-γ [44]. Furthermore, male patients are more prone to severe sepsis than female patients, just as males with sepsis present with mortality rates that are almost three times higher than females matched for age and disease severity [45,46,47]. A similar vulnerability ratio has been observed in male rodents, which are more susceptible to LPS-induced sepsis [48,49] and show higher levels of TNF compared to female animals [50]. Additionally, sickness behavior in this study developed faster in male WT rather than TNF-KO mice, suggesting that TNF is a central component of the acute response to LPS. These observations are in agreement with studies examining the time-course of LPS-induced effects on cytokine secretion [51], showing that TNF is increased earlier relative to other pro-inflammatory cytokines after LPS treatment, both in vitro [52] and in vivo [53]. Moreover, sickness behavior had largely dissipated 24 h post-LPS injection, a finding that is consistent with the time-course for the induction of TNF expression after the peripheral administration of LPS, as reported by this and other studies [1,54,55]. Collectively, our observations indicate that TNF is a major contributor to LPS-induced sickness behavior in mice, predominantly in male animals.

Based on the vulnerability of male subjects to the non-cognitive effects of LPS in our initial study, we conducted the BMT using male mice, in order to increase the probability of detecting effects of TNF deletion on spatial learning and memory. PBS-treated TNF-KO mice were impaired in the BMT during the acquisition and memory-probing phases of the test. These mice did not show a progressive reduction in total and primary escape latency during the acquisition phase and spent the lowest amount of time in the target block 24 h after the learning trials. The impaired performance of PBS-treated TNF-KO mice was also evident on probe DAY 4, when their nose-poking behavior in the BM was practically random. These findings are in good agreement with previous studies [2,7], and show that certain basal levels of TNF are required for spatial learning and memory in male mice.

However, it should be noted that cognitive impairment in TNF-KO mice has not been reported consistently in the literature [reviewed in 27], with several studies showing no effect [17,18,56] or even improvements [12] in spatial learning following the developmental deletion of TNF. While the exact reason for this inconsistency is unknown, it is possible that the nature of the cognitive task (BMT vs. MWMΤ), differences in protocols within tasks, or differences in the strain, sex and age of experimental animals [57], contribute to the discrepancy in results. Ageing, in particular, exerts complex effects on the regulation of spatial cognition. For example, memory deficits in the BMT were observed here in male, 11-13-month-old TNF-KO mice, but have not been reported in studies using male, 2-4-month-old TNF-KO mice [12]. Moreover, age-dependent improvements, rather than deficits, have been also reported with TNF deficient mice in the BMT [19]. Of relevance in the context of age-dependent TNF modulation, we previously showed that cortical TNF mRNA levels increase in female C57BL/6 × C3H/HeN mice from 21- through 24-month of age [13,58], and that cortical TNF protein levels increase with age in female C57BL/6J mice [59]. Moreover, TNF mRNA expression levels in microglia, the major source of TNF protein in the CNS, are higher in 24-month-old compared to 5-month-old mice [60]. It is thus plausible that the behavioral consequences of TNF deletion depend heavily on the age of the experimental animals, which may partly explain the inconsistent results obtained in the BMT or the MWMT using TNF-KO mice.

To examine how TNF deletion impairs spatial learning and memory in TNF-KO mice, the concentration of glutamate and noradrenaline was measured in the hippocampi of male mice. TNF can exert direct effects on glutamatergic [61] and noradrenergic neurotransmission [62], and both neurotransmitter systems are known to play important roles in learning and memory [63,64]. We observed no differences in the levels of glutamate and noradrenaline between KO and WT mice, indicating that gross disturbances in neurotransmitter homeostasis cannot account for the impaired BMT performance of TNF-KO mice. Future microdialysis studies might be useful in determining whether there are subtle differences in the extracellular concentration of glutamate and noradrenaline between genotypes.

Although the exact mechanisms by which TNF modulates cognition were not identified here, our results imply that other LPS-induced molecules can compensate for the effects of TNF on learning and memory. Compensatory effects for the absence of TNF were evident in LPS-treated TNF-KO mice, which showed no deficits in the BMT arena. Among the broad range of cytokines, chemokines and growth factors that are released upon LPS treatment, IL-1β could be a candidate for replacing the absence of TNF in TNF-KO mice. In IL-1 type 1 receptor knockout mice, TNF has been shown to mimic the effects of IL-1β on LPS-induced sickness behavior [65] and working memory [66], indicating that a certain degree of functional overlap exists between the two cytokines in response to LPS, at least in young adult mice. Interestingly, microglial mRNA [60] and cortical protein levels of IL-1β [59] show age-dependent increases in ageing mice, similar to what has been reported for TNF. 

The observation that constitutive levels of TNF are required for spatial cognition in male subjects, and the large body of evidence documenting sex differences in the immune response to peripheral inflammation, prompted us to study the effects of TNF deletion in the BMT using female subjects. Unlike male mice, no differences in spatial learning and memory were observed between female WT and TNF-KO mice, which is in line with previously reported results from our group, showing no effect of TNF deletion on baseline BMT performance in 9-month-old female mice [24]. These results indicate that sex differences in response to TNF deletion are not limited to LPS-induced sickness behavior but may extend to aspects of physiological cognitive function. Sex differences in response to TNF-inhibiting drugs have been repeatedly observed in chronic inflammatory diseases, with males being more responsive to non-selective anti-TNF treatment compared to females, even when controlling for baseline risk factors [67,68]. Several putative mechanisms have been proposed to explain how the immune system modulates learning and memory in a sex-dependent manner [30] including complex links between hippocampal cognition, neurogenesis, and circulating hormones [69]. 

In conclusion, the present data support a physiological role for TNF in modulating spatial learning and memory in male subjects. The effects of TNF deficiency were modulated by prior systemic inflammation, since LPS administration 48 h before the BMT reversed the deficits in TNF-KO mice, emphasizing the complexity and perhaps redundancy of inflammatory signaling in the brain. In addition, we note a close association between male gender and responsiveness to TNF, which is not limited to LPS-induced sickness behavior but may extend to aspects of physiological cognitive function. These results underline the importance of sex-stratified analysis when examining the role of TNF signaling in the brain and invite further consideration of gender as an important determinant of the response to TNF drugs.

### Limitations and Future Directions

The possibility that the effects of TNF deletion on cognition were masked by the higher motor activity in LPS-treated TNF-KO vs. WT male mice cannot be excluded by the present data. However, previous studies indicate that mouse motor behavior recovers within 48 h after the injection of LPS [70,71,72]. Moreover, differences in cognition between male TNF KO and WT mice were observed under baseline conditions, and there was no difference in total distance travelled between PBS-treated WT and TNF-KO mice. Therefore, any residual effects of LPS at the 48 h time-point do not affect the main conclusion of this manuscript, which is that baseline TNF is required for spatial cognition in male subjects.

The small sample size in the BM studies employing female TNF-KO mice and the possibility of a Type II error should also be noted as study limitations. The scarcity of 11-13-month-old female mice prevented us from studying the effects of LPS administration on cognition in both sexes. Such studies are warranted, given the reported sex differences in response to the endotoxin. Additionally, to control for genetic confounds comparing TNF-KO and WT mice it would be advisable to include heterozygous mice in future studies.

Additionally, systematic studies of microglial TNF expression in the aging male and female mice are required. Such studies ideally be performed at the single cell level, and include co-expression analysis of other cytokines, such as IL-1β, which we have previously shown to be expressed in a subset of microglia distinct from the TNF expressing microglia [14].

Finally, studies in mice with inducible, cell-specific deletion of TNF can be used to confirm whether microglia, the major source of TNF in the CNS, mediate the effects of TNF deletion on cognition in male subjects [13,14,16].

## Data Availability

Data is contained within the article.
